# Domestic violence and its predictors among married women in reproductive age in Fagitalekoma Woreda, Awi zone, Amhara regional state, North Western Ethiopia

**DOI:** 10.1186/1742-4755-10-63

**Published:** 2013-12-05

**Authors:** Agumasie Semahegn, Tefera Belachew, Misra Abdulahi

**Affiliations:** 1 School of Nursing and Midwifery, Haramaya University, Po Box 235, Harar, Ethiopia; 2 Department of Population and Family Health, Jimma University, Jimma, Ethiopia

**Keywords:** Domestic, Violence, Women, Ethiopia

## Abstract

**Background:**

Violence against women is one of the most systematic and prevalent human rights abuses in the world. It is a form of discrimination and deeply rooted in power imbalances and structural inequality between women and men. Documenting the extent of the problem and associated factors is essential to develop public health interventions to tackle violence against women. Therefore, the objective of this study was to determine magnitude of domestic violence and identify its predictors among married women in the reproductive age in north western Ethiopia.

**Methods:**

Community-based cross-sectional study was conducted from February 15 to March 15, 2011 among 682 married women and 46 key informants. Systematic sampling technique was used to select respondents for the quantitative method. Purposive sampling was used to select in-depth interview key informants for and focus group discussants. Data were analyzed using SPSS window version 16.0. Binary logistic regression and multivariable logistic regression analysis were carried out to determine the prevalence and identify independent predictors of domestic violence against women. Statistical association was measured by adjusted odds ratios and 95% confidence intervals. Statistical significance was declared at P < 0.05.

**Result:**

The prevalence of domestic violence was 78.0%. About 73.3%, 58.4% and 49.1% of women reported different forms of psychological, physical and sexual violence, respectively. Alcohol consumption by husband (AOR = 1.9, 95%CI = 1.3, 2.8), being pregnant (AOR = 2.1, 95% CI = 1.4, 3.4), decision making power (AOR = 2.3, 95% CI = 1.5, 3.4) and annual income (AOR = 1.9, 95% CI = 1.1, 3.3) were predictors of domestic violence.

**Conclusion:**

The prevalence of domestic violence was very high as compared to other studies. Women’s husband alcohol consumption, decision making power annual household income and being pregnant are some of the predictors of domestic violence against women.

## Background

Violence against women is one of the most systematic and prevalent human rights abuses in the world which is occurring in every continent and country that harms families, communities and reinforce inequality and violence [1]. Violence against women affects their productivity, autonomy, quality of life and physical and mental well being [2].

Domestic violence is a common phenomenon in Ethiopia both urban and rural families. About 81% of women believed that a husband is justified in beating his wife [3]. About 30-60% of families were affected by their intimates [4]. Domestic violence is the most far-reaching and prevalent. Women suffer physical, emotional, sexual and economic violence by their intimate partners. It is often protected by family secrecy, cultural norms, fear, shame, community’s reluctance on domestic affair and social stigma made the women remain silent [3,5,6].

The World Health Organization multi-country study indicated that worldwide domestic violence against women was ranged 15% in Japan to the highest level 71% in Ethiopia [6]. As a result, lack of land rights for women especially in rural areas, gender-gap in working areas, and access to education and health, widespread belief makes women inferior in Ethiopia. Ethiopia is a state party to many international and regional human rights instruments including the convention on the elimination of discrimination against women. The Ethiopian government recognizes the role played by civil society organizations in the promotion and protection of women’s rights [7]. However, violence against women is very common in Ethiopia even in the twenty first century.

Since this study conducted in the rural settings in which 84% of the Ethiopian population lives. This study investigated how much women were victim at home by their partner and what the contributing factors. This study would close the information gap regarding the current situation of domestic violence against women through revealing the magnitude of the problem. Therefore, this study was aimed to determine magnitude of domestic violence and identify its predictors among married women in the reproductive age in North Western Ethiopia. Determining the magnitude of domestic violence and indentifying predictors’ helps for government officials and any concerned bodies to design prevention and controlling to strategies to alleviate. Information obtained here can be used for different part of the country in similar setting.

## Methods

### Study setting

Community based cross-sectional study using both quantitative and qualitative methods of data collection was conducted in Fagitalekoma woreda, Awi zone, Amhara regional state of Ethiopia from February 15 to March 15, 2011. Fagitalekoma woreda (district) is one of the seven woredas found in Awi zone, Amhara regional state of Ethiopia and located 465 km from Addis Ababa and 105 km from Bahir Dar. The woreda had 25 rural and 2 urban kebeles (kebele or peasant association that is the smallest administrative unit that consist of 1000–1500 households). The woreda had an estimated total population of 174,876 of whom 86,619(49.53%) were males and 88,257(50.47%) were females. Majority (91.65%) population were rural dwellers.

### Study design and data collection

Community based cross-sectional study design was conducted between February 15 to March 15, 2011. Six hundred eighty two married women in the childbearing age were involved on the house to house interviewees. Forty six purposively selected participants were involved in the qualitative study. Then sample size was allocated proportionally to the size for five rural and two urban stratified kebeles (peasant associations). Finally, systematic sampling technique was used to select subjects. Four focus group discussions were employed on women that had 8 to 10 participants. Twelve in-depth interviews were conducted on key informants from the community representatives. The quantitative data were collected using a structured questionnaire adapted from WHO multi country studies on domestic violence against women and other studies done on intimate partner violence [3,6,8,9]. The questionnaire was contextualized to local situation and to research objectives. Local language (Amharic) version questionnaire and guide were used for data collection.

### Statistical analysis

To assure the quality of the finding, data collectors training and pre-test were conducted prior to the study had been conducted. The supervisors and principal investigator had checked the questionnaire each day. Incomplete and unclearly filled questionnaires were given back to the interviewer and got completed. Pre-coded data was entered and cleaned and analyzed using SPSS for windows version 16. Descriptive statistics were computed to determine prevalence of domestic violence and its predictors. Binary logistic regression analysis was also done to identify variables that are candidates for the multivariable analysis. Multivariable logistic regression analysis was carried out by taking significant variables in the binary analysis to control the confounding effect of other variables and to determine independent predictors of domestic violence against women. The statistical association was measured by AORs, 95% CIs and P < 0.05.

The qualitative data was transcribed by replaying the tape recorded interview. The text was thoroughly read, reread and thematic areas were identified. Ideas were colour coded and categorized under different thematic areas. Their inductive meanings were extracted and described in narratives using well said verbatim of the participants. The findings were presented triangulated with the quantitative data.

### Ethical review

The study was ethically approved by the ethical committee of college of public health and medical sciences at Jimma University. Verbal consent was obtained from each study participant. Confidentiality and the right of respondents not to participate were respected.

## Results

### General characteristics of study subjects

Six hundred eighty two married women were involved in the study yielding a response rate of 100%. The mean age of women was 31.6 (±7.5) years. Almost two third (65.4%) of women were Amhara ethnic. Majority (99.4%) of the respondents were orthodox Christians. five hundred ninety nine (87.8%) of them were rural dwellers. More than half (59.7%) of women were illiterate, and 552(80.9%) were housewives (Table 1). One fifth of the women’s husbands (20.2%) were in the age group 40–44 with the mean age of 40.8 (±10.8) years. More than one third (37.5%) of women’s husbands were illiterate and three fourth (76%) of them were farmers. More than two-third (68.9%) of husbands used drug in which alcohol took the highest share 465(98.9%). One hundred four (22.1%) husbands used alcohol very often while more than three quarters (77.9%) used it sometimes. More than one third (35.3%) of the women were pregnant during the last 12 months.

**Table 1:** Socio-demographic characteristics of married women in the reproductive age in Fagitalekoma Woreda, Awi zone, North Western Ethiopia, March, 2011 [n=682]

**Variables**		**Frequency**	**Percent**
Age of women	15-24	130	19.1%
	25-34	307	45.0%
	35-44	198	29.0%
	44+	47	6.9%
Ethnicity	Amhara	446	65.4%
	Agew	236	34.6%
Religion	Orthodox	678	99.4%
	Muslim	2	0.3%
	Protestant	2	0.3%
Educational status of women	Illiterate	407	59.7%
	Read and write	102	15.0%
	1-6 grades	80	11.7%
	7-12 grade	49	7.2%
	12+	44	6.5%
Residence	Rural	599	87.8%
	Urban	83	12.2%
Occupational status of women	House wife	552	80.9%
	Farmer	48	7.0%
	Merchant	14	2.1%
	Government employee	50	7.3%
	Private or NGOs employee	18	2.6%
Income contribution by women (USD)	<89.30	44	35.5%
	89.30-332.21	39	31.5%
	>332.21	41	33.1%

Majority (97.4%) of the households were headed by men. More than half (55.5%) of women had 1–4 alive children from current husband. Three hundred eighteen (46.6%) married women lived less than ten years with the current husband. Majority (97.1%) of women were formally married with current husband. The marital duration ranged from 1–36 years with mean marital duration of 12.7(±7.9) years. One-third (33.3%) of the household had annual income less than 280.22 US Dollar. Only 14(2.2%) women had independent decision making on large household purchases. Nearly half (48.7%) of women reported that they decided independently on small daily purchases. Women had joint decision making to visit their family and to use family planning were 72.7%, 69.2% respectively (Table 2).

**Table 2:** Distribution of decision making participations of married women on household issues in Fagitalekoma woreda, Awi zone, North Western Ethiopia, March, 2011 [n=682]

**Variables**		**Frequency**	**Percent**
Decision making on large purchases	Husband	383	56.2%
	Wife	14	2.2%
	Both	285	41.6%
Decision making on small daily purchase	Husband	80	11.7%
	Wife	332	48.7%
	Both	270	39.6%
Decision making to visit family or relatives	Husband	128	18.8%
	Wife	58	8.5%
	Both	496	72.7%
Decision making on FP utilization	Husband	134	19.6%
	Wife	76	11.1%
	Both	472	69.2%

### Domestic violence against married women

**Figure 1:**
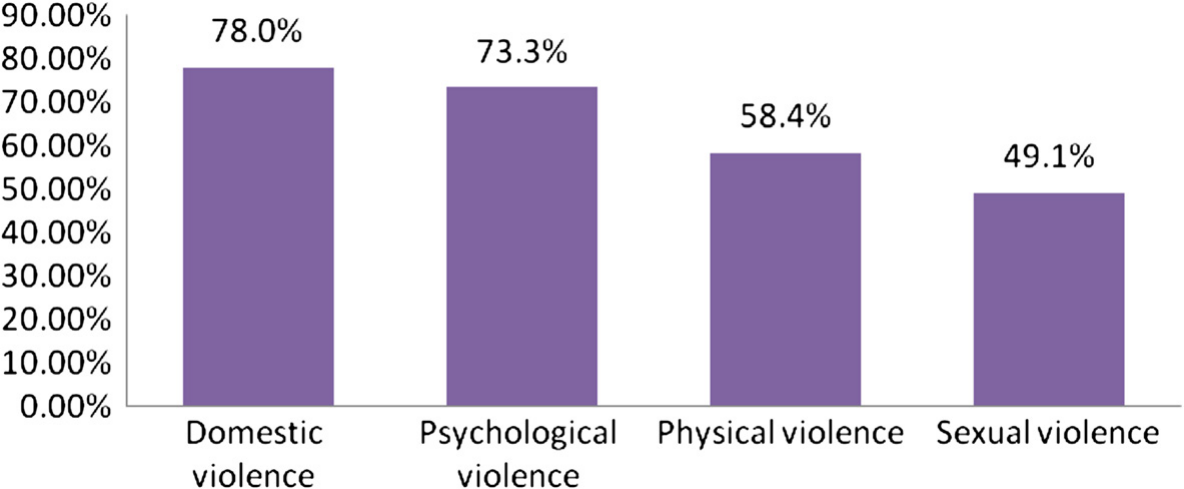
Distribution of domestic violence against married women in the reproductive age in Fagitalekoma woreda, Awi zone, North Western Ethiopia, March, 2011.

More than three quarter (78.0%) of married women reported that they had experienced at least one type of domestic violence by their current husband during the last 12 months. Two hundred fifty nine (37.9%) women reported psychological or physical or sexual violence attempted and or committed at the same time by their husband (Figure 1).

### Domestic psychological violence

Five hundred (73.3%) women reported different forms of psychological violence against them by their husband. Among these: women reported husband has jealous or angry if she talked to other men, insist on knowing where women were at all time, insult by using abusive language, threaten by an object like stick, belt, gun (other types of weapon), frightened by looking angrily at her, done things to scare or intimidate them purposely and restricted from visiting their parents or relatives and to attend workshops by their husband were 59.5%, 56.9%, 51.0%, 23.5%, 33.1%, 27.1% and 28.0% respectively (Table 3).

**Table 3:** Psychological violence among married women in the reproductive age in Fagitalekoma woreda, Awi zone, North Western Ethiopia, March, 2011

**Types of psychological violence**	**Frequency**	**Percent**
Jealous or angry if she talk to other men	406	59.5%
Insisted knowing where the women at all time	388	56.9%
Insulted by their husband using abusive language	348	51.0%
Threatened by an object like stick, belt, gun or other weapon	160	23.5%
Create financial hardship or not trust her	139	20.4%
Frightened their husband by looking angrily at her	226	33.1%
Expressed suspicion or accuses him that he is unfaithful	140	20.5%
Ignored or shown in difference	127	18.6%
Deprived privilege in the family	89	13.0%
Denied by their husband on their basic personal needs	94	13.8%
Intentionally makes not involved on decision making	95	13.9%
Belittled or humiliated them in front of others	87	12.8%
Done things to scare or intimidate them purposely	185	27.1%
Restricted from going to their parent’s, relative or workshops	191	28.0%

** Multiple response were possible.

### Domestic physical violence

Three hundred ninety eight (58.4%) women reported that they had experienced different forms of physical violence during the last 12 months by their husband. Among these women: 375(55.0%) reported being pushed, shaved, thrown something at her, 320(46.9%) reported being punched, hit with fist, twist her arm that could hurt her, 202(29.6%) reported being slapped, kick, dragged or beaten, 38(5.6%) reported being attacked with knife, gun and 31(4.5%) reported being ever scalded or burnt purposefully by their husband during the last 12 months (Table 4). Among those women who reported physical violence, 181(45.5%) of them had different outcomes. Such as 137(75.7%) had bruise or physical body aches and 41(22.7%) injured or broken bone. About 47(6.9%) women reported that their household properties were deliberately damaged by their husband during the last 12 months.

**Table 4:** Physical violence among married women in the reproductive age in Fagitalekoma woreda, Awi zone, North Western Ethiopia, March, 2011

**Types of physical violence**	**Frequency**	**Percent**
pushed, shaved, shake or throw something to her	375	55.0%
punched, hit with fist, twist their arm that could hurt her	320	46.9%
slapped, kicked, dragged or beaten	202	29.6%
attacked her with knife, gun, or other type of weapon	38	5.6%
ever scalded or burnt purposefully	31	4.5%

* Multiple responses were possible.

One of the key informants from women and children affair office stated woman’s history that came to seek help from their office *“…The husband beat his wife over the whole body…it was really terrible that makes me shocked. It was really shameful. He beat his wife by locking the door… Nobody could help her in their village. The reason was that her relative invited them for the welcome ceremony of her brother who was returning from army service. But he didn’t want to go there. Then she went to her relative and met her brother without her husband permission. She came back to her home at the same day even if she wanted to stay there to share their happiness… At that time the husband entered home and closed the door and started beating her. Oh! Really he is not human being….”*

### Domestic sexual violence

**Figure 2:**
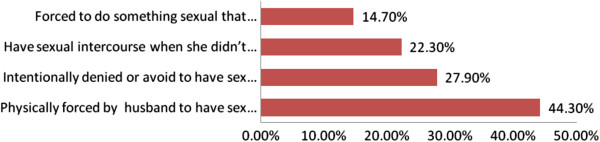
**Distribution of sexual violence among married women in the reproductive age in Fagitalekoma woreda, Awi zone, North Western Ethiopia, March, 2011.** [*Multiple responses were possible*].

Three hundred thirty five (49.1%) women reported that they had experienced different forms of sexual violence by their husband during the last 12 months. Among these women being physically forced to have sex when they didn’t want to have sexual intercourse, being intentionally denied sex and being forced to do something sexual that was degrading or humiliating her were 44.3%, 27.9% and14.7% respectively by their husband in one year time (Figure 2). The women reported their justifications to refuse having sex with their husbands; if she knew that her husband has sexually transmitted diseases 409(60.0%), if she knew that her husband has sexual relation with other women (66.3%) and if she is tired or has not in right mood 364(53.4%)*.* The women also reported their fears and expectations when they refused to have sex with their husband. Four hundred two (58.9%) women reported that he gets angry and reprimand her, 180(26.4%) reported that he give up financial support, 328(48.1%) reported that he can use force to have sex with her even if she doesn’t want to and 271(39.7%) reported that he goes to have sex with other women.

### Predictors of domestic violence

Age, ethnicity, residence, educational status, occupational status, women’s income contribution, age of husband, husband drug use, being pregnant, educational status of husband, occupational status of husband, head of household, number of children alive, marital duration, type of marriage, household annual income, decision maker of household were entered into binary logistic regression. Among these variables; educational status of women, occupational status of women, age of husband, husband drug use, being pregnant, household annual income and decision maker of household were significantly associated with domestic violence.

Variables like husband’s drug use, being pregnant, decision making power on household issues, age of husband and annual house hold income were positively associated with domestic violence against married women. Women whose husbands’ use alcohol were 1.9 times more likely to experience domestic violence than who do not use alcohol (AOR = 1.9, 95%CI = 1.3, 2.8). Women who were pregnant in the last 12 months were 2.1 times more likely to experience domestic violence than who were not pregnant (AOR = 2.1, 95%CI = 1.4, 3.4). Women living in a household earning annual income 280.22-508.76 US Dollar were 1.9 times more likely to experience domestic violence than women living in a household earning less than 280.22US Dollar [AOR = 1.9, 95%CI = 1.1, 3.3). Women whose husbands were decision makers of household issues were 2.3 times more likely to experience domestic violence than who made decisions jointly (AOR = 2.3, 95% CI = 1.5, 3.4) (Table 5).

**Table 5:** Multivariable logistic regression analysis show independent predictors of domestic violence among married women in the reproductive age in Fagitalekoma woreda, Awi zone, North Western Ethiopia, March, 2011

**Variables**		**Domestic violence**			
		**Yes n (%)**	**No n (%)**	**COR (95% CI)**	**AOR (95% CI)**
Husband drug use	Yes	362(72.4%)	107(58.8%)	1.9(1.3,2.8)	1.9(1.3,2.8)
	No	138(27.6%)	75(41.2%)	1.0	1.0
Pregnancy of women	Yes	202(40.4%)	39(21.4%)	2.1(1.4,3.2)	2.1(1.4,3.4)
	No	298(59.6%)	143(78.6%)	1.0	1.0
Decision maker of household issues	Husband	343(68.6%)	82(45.1%)	2.6(1.8,3.8)	2.3(1.5,3.4)
	Wife	10(2.0%)	5(2.7%)	1.3(0.4,4.3)	0.9(0.3,3.3)
	Jointly	147(29.4%)	95(52.2%)	1.0	1.0
Age of husband	20-24	7(1.3%)	9(6.0%)	1.0	1.0
	25-29	53(10.0%)	24(16.0%)	2.8(0.9,8.5)	3.4(1.0,11.2)
	30-34	99(18.6%)	26(17.3%)	4.9(1.7,14.4)	4.4(1.3,14.2)
	35-39	80(15.0%)	15(10.0%)	6.9(2.2,21.3)	6.7(1.9,23.4)
	40-44	108(20.3%)	30(20.0%)	4.6(1.6,13.5)	4.9(1.5,16.3)
	45-49	63(11.8%)	12(8.0%)	6.8(2.1,21.6)	7.2(1.9,26.5)
	≥50	122(22.9%)	34(22.7%)	4.6(1.6,13.3)	5.1(1.5,17.3)
Household annual income (USD)	<280.22	173(32.5%)	54(36.0%)	1.0	1.0
	280.22-508.76-	195(36.7%)	32(21.3%)	1.9(1.2,3.1)	1.9(1.1,3.3)
	>508.76	164(30.8%)	64(42.7%)	0.8(0.5,1.2)	0.7(0.4,1.2)
Educational status women	Illiterate	329(61.8%)	78(52.0%)	1.0	1.0
	Read and write	80(15.0%)	22(14.7%)	0.9(0.5,1.5)	0.8(0.5,1.5)
	1-6 grades	58(10.9%)	22(14.7%)	0.6(0.4,1.1)	0.5(0.3,0.9)
	7-12 grade	38(7.1%)	11(7.3%)	0.8(0.4,1.7)	2.0(0.9,4.9)
	12+	27(5.1%)	17(11.3%)	0.4(0.2,0.7)	1.3(0.5,3.1)

**Figure 3:**
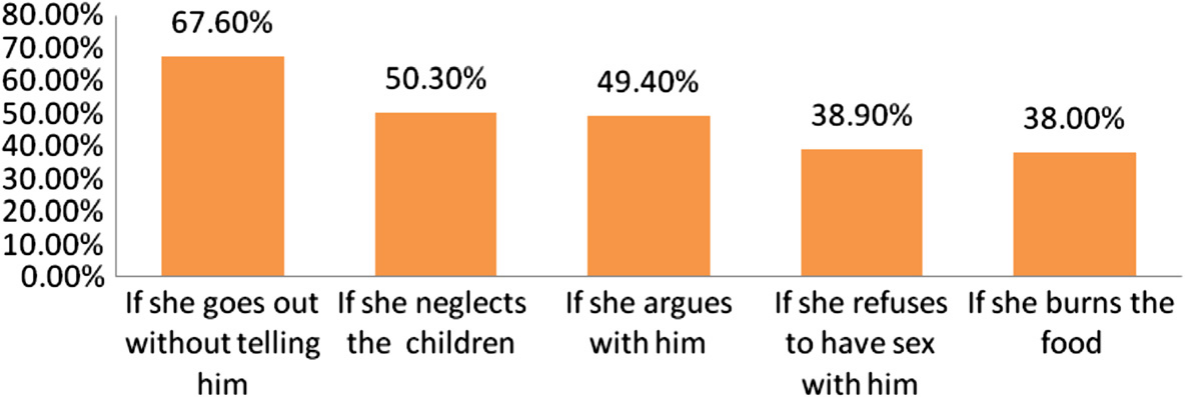
**Distribution of women’s reasons justified by husband for wife beat among married women (15–49 years) in Fagitalekoma woreda, Awi zone, North Western Ethiopia, March, 2011.** [*n = 682, multiple responses were possible*].

Among all respondents, 124(18.2%) women reported the presence of traditional gender norm that support wife beating. This was supported by qualitative data from different key informants for in-depth interview. A key informant from police said *“....mostly…the women themselves accept wife beating by the husband… the police arrived at a village during the conflict of the partners, the policeman has tried to manage the conflict and tried to bring the husband to kebele prison but the wife said to the policeman…why? You have no mandate to accuse him because he is my husband and he can kick me …*” Thirty six (5.3%) of women were accepting the traditional gender norm that support wife beating. Husband has the right to beat his wife if she goes out without telling him, if she neglect the children, if she argues with him, if she refuses to have sex with him and if she burns the food were 67.6%, 50.3%, 49.4%, 38.9%), 38.0% respectively (Figure 3).

## Discussion

This study determined the prevalence of domestic violence against women. The findings from this study showed that almost 8 out of every ten women were victim of domestic violence. Three fourth of women reported psychological violence and over half of the respondents were victim for different forms of physical and sexual violence by their partner within 12 months period. Husbands’ use alcohol, being pregnant, lack of decision making autonomy on household issues were more likely victim of domestic violence. This findings relatively consistent with WHO multi-country study on domestic violence showed that seven in every ten women (71%) had experienced domestic violence by their husband in Ethiopia. However, this finding is much higher than in Zambia almost half of women had domestic violence by their husband in Colombia, Peru, India and Dominican Republic, and also specific study done in Ethiopia (Gondar zuria district) almost half of women had domestic violence by their husband [6,10]. The possible reason might be the presence of traditional gender norm that support wife beating in the study area. Nevertheless, this is inlined with a meta-analysis from demographic and health surveys of 17 countries in sub-Saharan Africa indicated that domestic violence revealed that 74% in Ethiopia, and a study done in Nigeria showed one third of women victimized during their pregnancy period [11,12]. The psychological violence is quite higher than when compared with a study done in different countries in the world such as Brazil, Hog Kong, Maldives, Uganda, Ethiopia and South Africa [8,13-17]. This might be due to traditional gender norm that support men superiority in the study area and majority of the women had lived in rural area which is the problem mostly common and deep rooted.

Concerning about the different forms of physical violence against them by their husband, this finding is inlined with a study in rural Ethiopia (Meskan and Mareko) revealed that half of women (49.5%) had experienced physical violence by their partner [16]. Nevertheless, it is quite higher than when it compared with a study conducted in 8 South African countries indicated that partner physical violence ranged lowest rates from 9% in Mozambique and Malawi to the highest (32%) in Zambia [18]. It has significant difference as compared to study finding from South Africa, Japan (Yokohama) and in Brazil revealed that 1–4 in every 10 women had experienced physical intimate partner violence at some point in their past [13,17,19]. The difference might be presence of culture and traditional gender norms that support wife beating within the community. However, it is quite higher as compared with a study done in Maldives, Uganda and Tanzania showed that 11.4% to 24.8% of women had reported physical violence by their partner [8,15,20]. It has higher than findings from other studies done in different parts of Ethiopia (Gondar zuria district, kofale in Arsi zone and Agaro) indicated that a round one third of women had experienced physical violence by their partner [9,10,21]. The higher prevalence might be due to the study time difference in which in the current study victims may disclose more about the occurrence of the problem.

Regarding to sexual violence, the most frequent type of sexual violence reported by women was physically forced to have sex when they didn’t want to have sexual intercourse. This is incomparable higher than findings from a study conducted in Ethiopia (Gondar zuria district and Agaro) found that approximately one in five of women have been forced to sex by their partners [9,10]. Women were forced to do something sexual that degrade or humiliate them that was quite higher than a finding from study done in Turkey and in Brazil found that 3% and 9.8% women had experienced sexual violence respectively [13,22]. The observed difference might be due to sexual autonomy imbalance. In the qualitative study finding most of the focus group discussants agreed that**“**…*Husband has the right to beat his wife… if she refuses to have sexual intercourse with her husband …even unhealthy condition of women…”so* women didn’t want to refuse having sexual intercourse.

Those women whose husbands drink alcohol were more likely to experience domestic violence than their counterparts. Similarly different studies found that the occurrence of domestic violence doubled when the husband drink alcohol [10,14,23-25]. As the age of husbands going older the occurrence of domestic violence increases. This is contradicted with a study done in Tanzania, which indicated women who had older partners were less likely victim of violence than women who had younger partners [20]. Similarly a study done in Serbia and Vietnam showed that domestic intimate partner violence was doubled when the partner had only secondary education or below secondary education [25,26]. On the other hand, in this study women’s education is negatively associated with domestic violence which contradicts with a study in South African, Zimbabwean and Tanzania [4,20]. This might be as women’s’ educational level increase their awareness and struggle to their right against the traditional gender norm in which in this study population the elders were more accept the wife beating.

Women living in a household middle level annual income were more likely to experience domestic violence than the lowest annual income. This contradicts with the finding in India; higher family income was negatively associated with domestic violence [23]. Household’s low socioeconomic status in comparison to high was significantly associated with intimate partner violence [25]. The reason might be the income by itself creates disagreement that leads for straggle for ownership and decision making. Women whose husbands were decision makers of household issues were more likely to experience domestic violence than women who made decisions jointly with their husbands. This finding is similar with a study in Gondar zuria district [10]. Among domestic violence victims less than one third of women sought help concerned bodies. It is higher than a study in Turkey in which only 11.7% sought help after being exposed to physical violence [22]. This relative difference might be due to the availability of locally arranged conflict management committee from local leaders, elders, religious fathers and model families assigned by the community and woreda administration office.

### Implication of the findings

Although Ethiopia is a state party to many international and regional human rights instruments including the convention on the elimination of discrimination against women. Violence against women is a major obstacle to the progress in achieving growth and development targets, in which it recognize as a public health and human rights concern in Ethiopia. Therefore, determining the magnitude and indentifying predictors’ helps for government officials and any concerned bodies to design prevention and controlling strategies to tackle domestic violence. Preventing violence against women will contribute to the achievement of MDG number 3 that specifically addresses promotion of gender equality and women's empowerment. In addition, assuring gender equality is recognized as key in achieving all eight goals. Information obtained here can be used for planning of intervention programs in different part of the country.

### Strength and limitation of the study

This study is community based study which is mainly in rural setting that domestic violence is very common related to the presence of traditional gender norm that support wife beating. It has qualitative study that used to explore community perception about domestic violence mainly wife beating. However, it has limitations that it was based on self-reporting, there might be recall and social desirability bias due to its’ sensitive nature and cultural barrier for disclosure. This study did not include men to assess their attitude towards wife beating on quantitative study and the violence against men by their female partner.

## Conclusion

This study showed that domestic violence against women was quite high. Almost 8 out of every 10 women had domestic violence by their partner. Approximately three fourth of women were victim of psychological violence as well as more than half of the women had different forms of physical and sexual violence in the last 12 months by their husband. Husbands’ drink alcohol, being pregnant; lack of women decision making autonomy and annual income of household was more likely victim of domestic violence. Only one out of three victim women sought help mainly from their family who are highly tied by traditional gender norm that support wife beating. Therefore, we recommend that creating awareness to avoid of traditional gender norm that support wife beating in the community by integrating with health extension program to prevent domestic violence.

## Competing interests

All authors declare that they have no competing interests.

## Authors’ contributions

AS had made substantial contributions to conception and design, or acquisition of data, or analysis and interpretation of data, and TB and MA had revised the paper critically for important intellectual content. All authors read and approved the final manuscript.
